# Pressure-induced Pb–Pb bonding and phase transition in Pb_2_SnO_4_

**DOI:** 10.1107/S205252062001238X

**Published:** 2020-11-03

**Authors:** Dominik Spahr, Michał Stękiel, Dominik Zimmer, Lkhamsuren Bayarjargal, Katja Bunk, Wolfgang Morgenroth, Victor Milman, Keith Refson, Dominik Jochym, Peter J. P. Byrne, Björn Winkler

**Affiliations:** a Goethe University, Institute of Geosciences, Crystallography, Frankfurt, Germany; b Dassault Systèmes BIOVIA, Cambridge, United Kingdom; c Royal Holloway, University of London, Physics, Oxford, United Kingdom; d Scientific Computing Department, Rutherford Appleton Laboratory, Oxford, United Kingdom; e University of York, Physics, Heslington, United Kingdom

**Keywords:** lead stannate (Pb_2_SnO_4_), density functional theory, high-pressure X-ray diffraction, pressure-induced phase transition, insulator–semiconductor transition

## Abstract

High-pressure single-crystal to 20 GPa and powder diffraction measurements to 50 GPa, show that the structure of Pb_2_SnO_4_ strongly distorts on compression with an elongation of one axis. A structural phase transition occurs between 10 GPa and 12 GPa, with a change of space group from *Pbam* to *Pnam*. The resistivity decreases by more than six orders of magnitude when pressure is increased from ambient conditions to 50 GPa. This insulator-to-semiconductor transition is accompanied by a reversible appearance change from transparent to opaque. Density functional theory-based calculations show that at ambient conditions the channels in the structure host the stereochemically-active Pb 6*s* ^2^ lone electron pairs. On compression the lone electron pairs form bonds between Pb^2+^ ions. Also provided is an assignment of irreducible representations to the experimentally observed Raman bands.

## Abstract 2

The structure of Pb_2_SnO_4_ is found to strongly distort on compression and a structural phase transition with a change of space group from *Pbam* to *Pnam* occurs at ∼11 GPa. Our complementary DFT-based calculations show that at ambient conditions, the channels in the structure host the stereochemically active Pb 6*s* ^2^ lone electron pairs which form bonds between the Pb^2+^ ions with increasing pressure.

## 1. Introduction

Lead stannate (Pb_2_SnO_4_) belongs to a family of stannates with composition *M*_2_SnO_4_, where *M* ^2+^ = Mg, Mn, Ca, Ba, Sr, Pb. Characteristic for these stannates is that the SnO_6_ octahedra either form layers by sharing corners in a plane (as in Ba_2_SnO_4_ and isostructural Sr_2_SnO_4_) or chains by sharing edges (as in Ca_2_SnO_4_ and Pb_2_SnO_4_). They represent a fascinating class of compounds and have been studied extensively as they may be suitable for a variety of applications, *e.g.* as photocatalysts (Qin *et al.*, 2015 ▸; Dinesh *et al.*, 2016 ▸), as electrode material for Li-ion batteries (Rong *et al.*, 2006 ▸; Liang *et al.*, 2016 ▸) or as anode-material in solar cells (Tan *et al.*, 2007 ▸). Furthermore, stannates doped with rare earth elements, such as Eu, Y, Sm, have been extensively investigated regarding their use as long afterglow phosphors (Chen *et al.*, 2005*a* ▸,*b* ▸; Yang *et al.*, 2005 ▸; Yamane *et al.*, 2008 ▸; Zhang *et al.*, 2010 ▸; Stanulis *et al.*, 2014 ▸).

**Figure 1:**
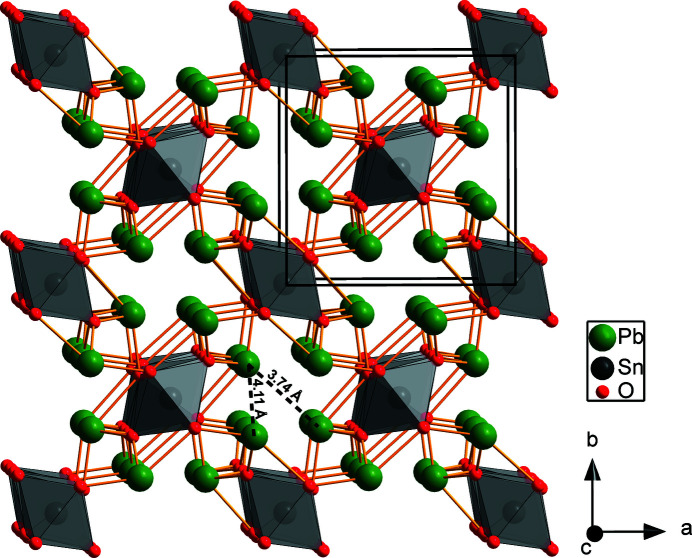
Structure of Pb_2_SnO_4_ at ambient conditions from single-crystal structure solution. A 2 × 2 × 2 supercell is shown along the *c* axis. The SnO_6_ octahedra are shown in gray. A diagonal (3.74 Å) and a line between two opposite Pb^2+^ ions (4.11 Å) in one of the channels which run along the *c* axis.

Pb_2_SnO_4_ has been used since the 14th century as a pigment and was frequently used in oil paintings before 1750. Nowadays the pigment is named lead-tin-yellow type I [see summary by Kühn (1993 ▸)]. Its structure at ambient conditions (Fig. 1 ▸) was first proposed to have tetragonal space group symmetry *P*4_2_/*mbc* (Byström & Westgren, 1943 ▸; Swanson *et al.*, 1972 ▸). Later, the structure has been described in the orthorhombic space group *Pbam* (Gavarri *et al.*, 1981 ▸).

In Pb_2_SnO_4_ the edge-sharing SnO_6_-octahedra form chains along the *c*-direction, interconnected within the (001) planes with Pb^2+^ ions. There are channels parallel to the *c* axis with a diameter of ≈ 3.74 Å (Fig. 1 ▸). In Pb_2_SnO_4_ the Pb ions form the apex of a trigonal pyramid, *i.e.* there are only three short Pb—O distances. This points towards the presence of a stereochemically active lone electron pair. In contrast, in Ca_2_SnO_4_, where edge-sharing SnO_6_ octahedra also form chains, the Ca ions have seven nearest neighbors forming an irregular polyhedron.

At ambient conditions, the structure of Pb_2_SnO_4_ closely resembles that of the mineral minium (Pb_2_PbO_4_), which crystallizes at ambient conditions in the tetragonal space group *P*4_2_/*mbc*. In Pb 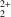 Pb^4+^O_4_, the Pb^4+^ atoms are octahedrally coordinated. Pb_2_PbO_4_ has channels with a diameter of ∼3.8 Å (Gavarri & Weigel, 1975 ▸). Pb_2_PbO_4_ undergoes a temperature-induced phase transition below 170 K to space group *Pbam* (Gavarri *et al.*, 1978 ▸), so that Pb_2_SnO_4_ and Pb_2_PbO_4_ are isostructural at low temperatures, as the former shows no temperature-induced phase transition between 5 K and 300 K (Gavarri *et al.*, 1981 ▸). For Pb_2_SnO_4_ the deviation from tetragonal symmetry remains small in this temperature range Δ*ab* = *a* − *b* = 0.0125 (6) Å at 300 K, Δ*a* *b* = 0.0139 (6) Å at 5 K].

Dinnebier *et al.* (2003 ▸) found that Pb_2_PbO_4_ undergoes a pressure-induced phase transition from space group *P*4_2_/*mbc* at ambient pressure to space group *Pbam* between 0.11 and 0.3 GPa and a second transition between 5.54 and 6.58 GPa to another orthorhombic phase, also having space group *Pbam* but with half the length of the *c* axis. Increasing pressure leads to an anisotropic compression of the *a* and *b* axes, with Δ*ab* ∼ 0 Å at ambient conditions to Δ*ab* ∼ 0.9 Å at 0.6 GPa and Δ*ab* ∼ 2.9 Å at 6.7 GPa.

The objective of this study was to characterize pressure-induced changes in structure–property relations of Pb_2_SnO_4_ at high pressures, as it was expected that by analogy with Pb_2_PbO_4_ the compound would undergo phase transitions and offer insight into the high-pressure behavior of stereochemically active lone electron pairs.

## 2. Experimental details

### 2.1. Sample synthesis

#### 2.1.1. Solid-state synthesis

Temperatures between 923 K and 1173 K have been used for the synthesis of Pb_2_SnO_4_ powder by solid-state reaction (Gavarri *et al.*, 1981 ▸; Hashemi *et al.*, 1992 ▸; Clark *et al.*, 1995 ▸; Chen *et al.*, 2000 ▸; Hradil *et al.*, 2007 ▸; Pelosi *et al.*, 2010 ▸; Denisov *et al.*, 2012 ▸; Agresti *et al.*, 2016 ▸). For our experiments we chose a synthesis temperature of 1123 (1) K, in order to avoid the presence of PbSnO_3_ which decomposes above 1073 K and to prevent decomposition of Pb_2_SnO_4_ which is expected to occur above 1173 K (Xing *et al.*, 2004 ▸).

The starting materials were analytical grade and used as purchased: PbO (99.9+% purity, Sigma-Aldrich Chemie, Darmstadt) and SnO_2_ (99.9% purity, Alfa Aesar, Karlsruhe). Starting materials were mixed in stoichiometric proportions and ground in an agate mortar. The resulting mixture was pressed to 5 mm-sized pellets with an Across International Desktop pellet press at a pressure of 6 (1) Kbar. The pellets were placed in corundum crucibles with lids, transferred into a Nabertherm L08/14 muffle furnace, heated up to 1123 (1) K and annealed for 24 h. The samples were cooled down to ambient temperature by switching off the power supply. After the synthesis the pellets were ground again and the synthesis process was repeated two times.

#### 2.1.2. Hydrothermal synthesis

Pb_2_SnO_4_ single crystals were hydrothermally grown in autoclaves according to a prescription by Wu *et al.* (1999 ▸), following the reaction

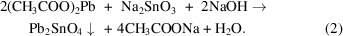

We used analytical grade Pb(CH_3_COO)_2_·3H_2_O (99.5% purity, Merck, Darmstadt), Na_2_SnO_3_·3H_2_O (98% purity, Alfa Aesar, Karlsruhe) and NaOH (99% purity, Merck, Darmstadt). The starting materials were dissolved separately in double distilled water to obtain solutions with 0.34 mol l^−1^ [Pb(CH_3_COO)_2_], 0.16 mol l^−1^ (Na_2_SnO_3_) and 2.0 mol l^−1^ (NaOH) concentration. First the Na_2_SnO_3_ and subsequently the NaOH solution were added dropwise to the Pb(CH_3_COO)_2_ solution while continuously stirring at ambient temperature. The resulting suspension was transferred into a 60 ml Teflon cup which was filled up to 60% of its volume and was then placed in stainless steel autoclaves. The autoclaves were heated up to 503 (1) K for 48 h, afterwards they were slowly cooled down to 298 (1) K within 72 h. The precipitate was recovered by vacuum filtration, washed with distilled water repeatedly and dried at 333 (1) K in an oven.

### 2.2. Sample characterization

#### 2.2.1. X-ray powder diffraction at ambient pressure

The powder samples obtained from solid-state synthesis were characterized with a PANalytical X’Pert Pro powder diffractometer with Bragg–Brentano geometry and a PANalytical PIXcel^3D^ detector. The diffractometer was equipped with a copper X-ray tube and a Johansson monochromator. The measurements were performed using Cu *K*α1 radiation and 0.25° fixed divergence slits. The samples were measured in the range of 10° < 2θ < 90° with a scan speed of 0.036° min^−1^. The instrument parameters were refined using a measurement on a high purity (99.999%) Si-standard. Powder samples were mounted on an oriented Si single-crystal sample holder after grinding them in an agate mortar. Crystal structure refinements, based on the Rietveld method (Rietveld, 1969 ▸), were carried out using the software package *GSAS-II* (Toby & Von Dreele, 2013 ▸).

#### 2.2.2. X-ray single-crystal diffraction at ambient pressure

Hydrothermally synthesized crystals were employed for the single-crystal diffraction experiments at ambient conditions. Measurements were carried out on an Oxford Instruments Xcalibur four-circle diffractometer with Kappa geometry and a Sapphire3 charge-coupled-device (CCD) detector. The diffractometer was equipped with a molybdenum X-ray tube and graphite single-crystal monochromator. The samples were measured with Mo *K*α radiation. We measured a full sphere up to a resolution of 0.75 Å^−1^ and an exposure time of 120 s per frame. The crystals were mounted with Apiezon N grease on the tip of a glass capillary. Crystals of approximate dimensions 80 µm × 30 µm × 30 µm were centered in an X-ray beam of diameter 500 µm. The reflections were indexed and integrated using the *CrysAlisPRO* (v. 39.46) program (Agilent, 2014 ▸). The structure solution was performed with *SUPERFLIP* (Palatinus & Chapuis, 2007 ▸) and the refinement with the software package *JANA2006* (Petricek *et al.*, 2014 ▸).

#### 2.2.3. High-pressure experiments

All high-pressure experiments were carried out using Boehler–Almax-type diamond anvil cells (Boehler, 2006 ▸). Depending on the target pressure of the experiments we used culet sizes between 250 µm and 350 µm and tungsten or rhenium as gasket material. Samples were placed in holes in the gasket having diameters between 100 µm and 180 µm. The gaskets were pre-indented to 40–50 µm and the holes were drilled by a custom-built laser setup. We used argon below 3 GPa for the powder diffraction and neon for all other experiments as pressure-transmitting media in pressure ranges where they provide a quasi-hydrostatic environment (Klotz *et al.*, 2009 ▸). Pressure was determined by measuring the ruby fluorescence shift. We assume an error of 2% for the pressure determination in the quasi-hydrostatic conditions present in our experiments (Dewaele *et al.*, 2004 ▸, 2008 ▸).

#### 2.2.4. High-pressure synchrotron X-ray diffraction

High-pressure diffraction data were collected at the synchrotron PETRA III (DESY) in Hamburg, Germany on the extreme conditions beamline P02.2 (Liermann *et al.*, 2015 ▸). We used a Perkin Elmer XRD1621 detector and wavelengths of 0.2887 Å and 0.2906 Å for data acquisition. The beam size was 2 µm (H) × 2  µm (V) (FWHM) obtained using a Kirkpatrick–Baez mirror for the powder diffraction experiments and 9 µm (H) × 3 µm (V) (FWHM) obtained using compound refractive lenses for the single-crystal diffraction measurements.

The powder samples were measured for 10 s while rotating them around a rotation axis perpendicular to the beam by ±10° to improve the counting statistics. For calibrating the detector parameters and the detector to sample distance we measured a CeO_2_ powder standard. We used the program *DIOPTAS* (Prescher & Prakapenka, 2015 ▸) to integrate and calibrate the diffraction patterns.

For single-crystal diffraction the samples were rotated around a rotation axis perpendicular to the beam by ±33°. Frames were collected in 0.5° steps with 0.5 s acquisition time per frame. Pt-filters were used to reduce the primary intensity to prevent oversaturation of the detector. The diffractometer/detector geometry was calibrated by measuring an enstatite single crystal. Data treatment and crystal structure refinement were performed in a similar manner as for ambient-pressure single-crystal diffraction data.

The lattice parameters from the high-pressure powder diffraction data were obtained applying the Le Bail method (Le Bail *et al.*, 1988 ▸), using the software package *GSAS-II* (Toby & Von Dreele, 2013 ▸). The bulk modulus from the high-pressure powder data was obtained by using the *EoSFit7-GUI* software package (Gonzalez-Platas *et al.*, 2016 ▸), fitting a second-order Birch–Murnaghan equation of state (EoS) (Murnaghan, 1944 ▸; Birch, 1947 ▸) to the unit-cell volume.

#### 2.2.5. High-pressure electrical resistance measurements

**Figure 2:**
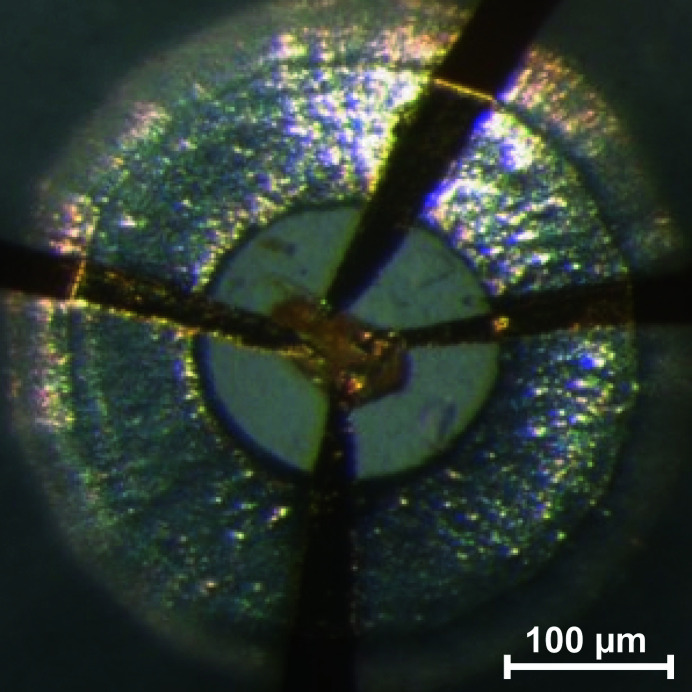
Pb_2_SnO_4_ crystal connected to gold wires in a diamond anvil cell for resistance measurements using the four-point probes method at 4.3 (3) GPa.

High-pressure resistance measurements were carried out in diamond anvil cells (Fig. 2 ▸) using a mixture of epoxy resin and Al_2_O_3_ as pressure-transmitting medium. We assume an error of the pressure determination due to non-hydrostatic conditions of 6% (Mao *et al.*, 1986 ▸). We performed both two-point and four-point measurements using a Keithley DMM7510 multimeter for the data collection as described in Zimmer *et al.* (2018 ▸).

#### 2.2.6. Raman spectroscopy

Raman spectroscopy was carried out with a custom set-up described in detail in Bayarjargal *et al.* (2018 ▸). We used an OXXIUS S.A. LaserBoxx LMX532 laser (λ = 532.14 nm) and a Princeton Instruments ACTON SpectraPro 2300i spectrograph equipped with a Pixis256E CCD camera. All Raman spectra were background corrected with the software package *Fityk* (Wojdyr, 2010 ▸). High-pressure Raman spectroscopy was carried out in diamond anvil cells, similar to the high-pressure synchrotron X-ray diffraction experiments.

#### 2.2.7. Scanning electron microscopy

We used a Phenom World ProX desktop SEM for the acquisition of electron backscatter images (BSE) on single crystals and powder samples. Furthermore, energy-dispersive X-ray spectroscopy (EDX) measurements for a semi-quantitative characterization of the composition were carried out on single crystals and powder samples. The samples were mounted without coating on aluminium stubs using sticky carbon tape. They were measured under low-vacuum conditions to reduce charging effects on the sample with an acceleration voltage of 10 KV for imaging and 15 KV for EDX measurements.

## 3. Computational details

First-principles calculations were carried out within the framework of density functional theory (DFT) (Hohenberg & Kohn, 1964 ▸), employing the Perdew–Burke–Ernzerhof (PBE) exchange-correlation function (Perdew *et al.*, 1996 ▸) and the plane wave/pseudopotential approach implemented in the CASTEP (Clark *et al.*, 2005 ▸) simulation package. ‘On the fly’ norm-conserving or ultrasoft pseudopotentials generated using the descriptors in the CASTEP data base were employed in conjunction with plane waves up to a kinetic energy cutoff of 990 eV or 630 eV, for norm-conserving and ultrasoft pseudopotentials, respectively. The accuracy of the pseudopotentials is well established (Lejaeghere *et al.*, 2016 ▸). A Monkhorst–Pack (Monkhorst & Pack, 1976 ▸) grid was used for Brillouin-zone integrations with a distance of < 0.023 Å^−1^ between grid points. Convergence criteria included an energy change of < 5 × 10^−6^ eV atom^−1^ for scf-cycles, a maximal force of < 0.008 eVÅ^−1^, and a maximal component of the stress tensor < 0.02 GPa. Phonon frequencies were obtained from density functional perturbation theory (DFPT) calculations. Raman intensities were computed using DFPT with the ‘2*n*+1’ theorem approach (Miwa, 2011 ▸) and a scissor operator of 5 eV. It should be stressed that all calculations are carried out in the athermal limit, *i.e.* the influence of temperature and zero-point motion is not taken into account.

## 4. Results

### 4.1. Synthesis

**Figure 3:**
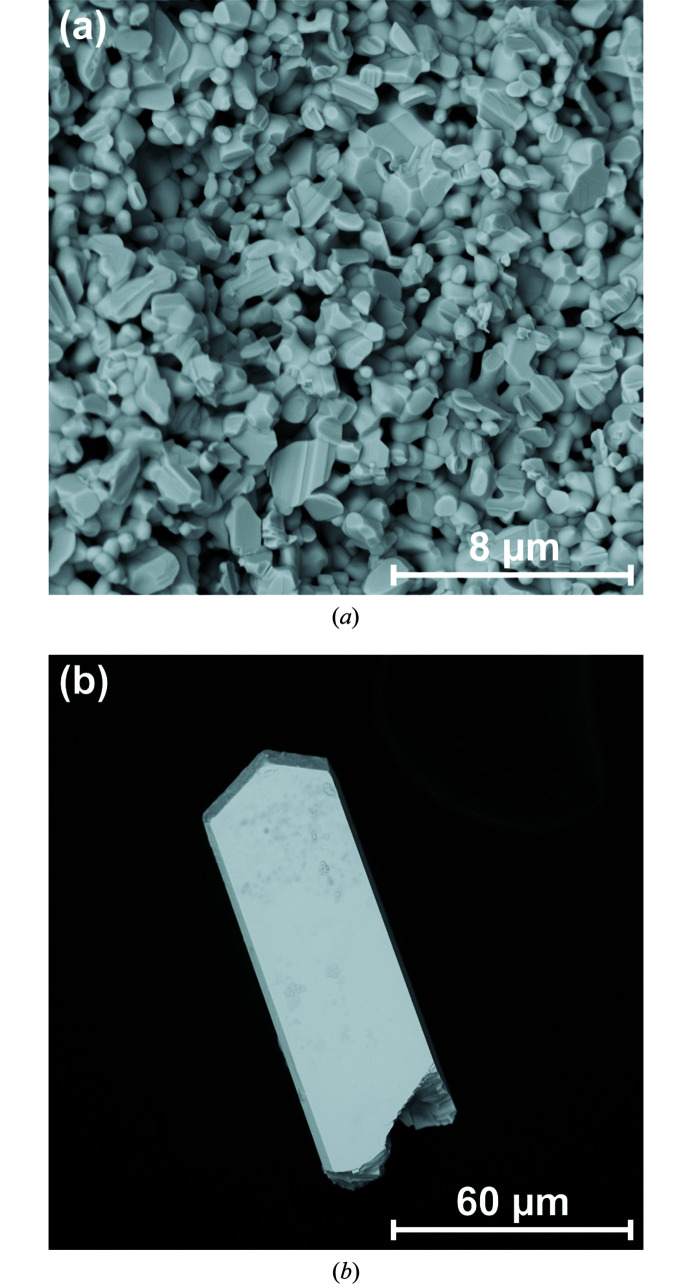
SEM image of slightly yellow Pb_2_SnO_4_ powder obtained by solid-state reaction (*a*) and of a colorless Pb_2_SnO_4_ crystal from hydrothermal synthesis (*b*).

We synthesized Pb_2_SnO_4_ powder by solid-state reaction. The powder is slightly yellow at ambient conditions and does not show any distinct growth morphology [Fig. 3 ▸(*a*)]. From hydrothermal synthesis we obtained crystals with 50–150 µm lengths. Some crystals form aggregates, but many of them show a tetragonal crystal habit and idiomorphic crystal faces [Fig. 3 ▸ *b*]. Most of those crystals are colorless. The morphology of the single crystals is similar to that observed in an earlier study (Wu *et al.*, 1999 ▸).

The chemical composition obtained from the EDX measurements on the powders and single crystals do not substantially differ from the expected chemical composition (nominal versus EDX in at.%): PbO: 67/70 (4) and SnO_2_: 33/30 (3) for the powders from the solid-state reaction and PbO: 67/71 (4) and SnO_2_: 33/29 (3) for the single crystals from the hydrothermal synthesis. Furthermore, no impurities from other elements were observed in the EDX spectra.

### 4.2. Powder diffraction at ambient pressure

**Figure 4:**
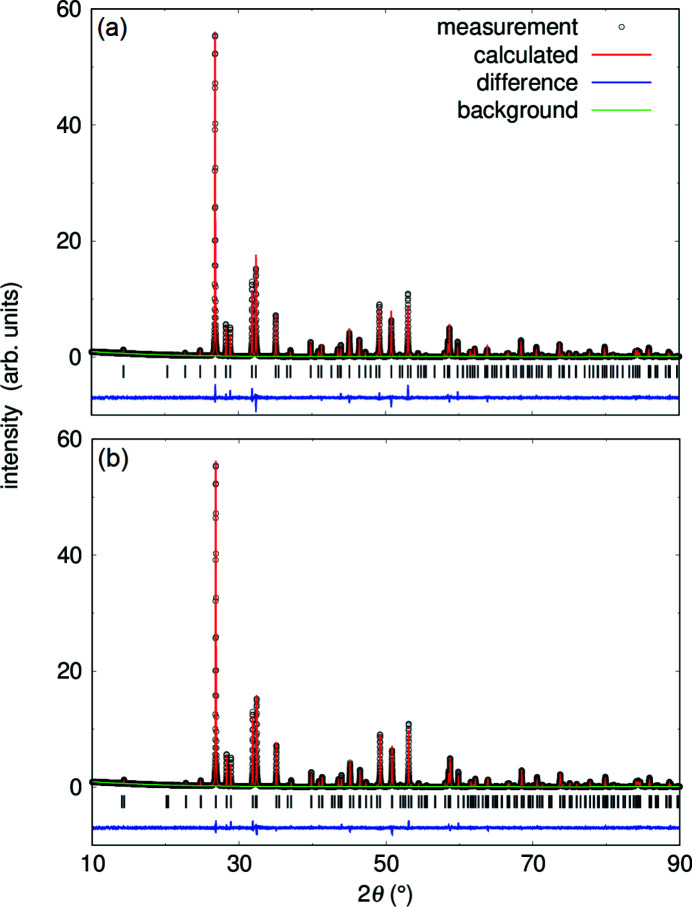
Rietveld refinements on a Pb_2_SnO_4_ powder sample from solid-state synthesis at ambient conditions in space group *P*4_2_/*mbc* (*a*) and *Pbam* (*b*) using λ = 1.54056 Å. Reflection positions are indicated by tickmarks and the residuals between measurement and refinement are shown by the blue line.

No secondary phase was detected by X-ray powder diffraction within the experimental detection limits of ∼3%. The phase purity of the powder was also confirmed by Rietveld refinement (Fig. 4 ▸). The refinement of the powder data can be carried out in two space groups. A refinement in the tetragonal space group *P*4_2_/*mbc* (*wR* = 12.1%) as well as in its orthorhombic subgroup *Pbam* (*wR* = 10.7%) with *Z* = 4 gave a satisfactory fit of the structural model to the diffraction data. Table 1 ▸ summarizes the diffraction data for Pb_2_SnO_4_ at ambient conditions. The refined crystallographic parameters for both space groups are also in good agreement with earlier studies (Swanson *et al.*, 1972 ▸; Gavarri *et al.*, 1981 ▸).

The deviation from a tetragonal metric in the orthorhombic refinement [Δ*a* *b* = 0.0095 (3) Å] is very small and in good agreement with the value obtained by neutron powder diffraction data from Gavarri *et al.* (1981 ▸) [Δ*a* *b* = 0.0125 (6) Å]. Furthermore, no peak splitting was observed in the diffraction data. The refinement in space group *Pbam* shows a slightly lower *wR* value compared to the refinement in space group *P*4_2_/*mbc*. This is caused by the higher reflection to parameter ratio (6.5:1 for *Pbam* in contrast to 4.5:1 for *P*4_2_/*mbc*).

### 4.3. Single-crystal diffraction at ambient pressure

The structure solution for Pb_2_SnO_4_ by Gavarri *et al.* (1981 ▸) was performed on neutron powder data; here we carried out the first single crystal data collection and structure refinements. High-quality crystals from our hydrothermal synthesis are colorless at ambient conditions and suitable for single-crystal X-ray diffraction.

Similar to the results obtained from the powder diffraction data at ambient conditions, the refinement of the single crystal data gives very satisfactory results in two space groups. Both structure refinements in space group *P*4_2_/*mbc* (*wR* = 2.3%) and in space group *Pbam* (*wR* = 2.6%) gave convincing structural models (Table 1 ▸), which are in good agreement with the results of earlier studies (Swanson *et al.*, 1972 ▸; Gavarri *et al.*, 1981 ▸). Table 2 ▸ summarizes the atomic positions and anisotropic displacement parameters of Pb_2_SnO_4_ for the refinements in both space groups.

The anisotropic displacement parameters do not differ substantially between the structure refinements in space group *P*4_2_/*mbc* and *Pbam*. Lowering of the symmetry from *P*4_2_/*mbc* to *Pbam* space group induces a splitting of the Wyckoff positions of the Pb1 and O1 atoms with 8*h* → 4*g* + 4*h*, but a detailed analysis showed no significant change in interatomic distances between the two refinements.

### 4.4. DFT calculations at ambient pressure

We cross-checked our experimental results with those of DFT-based calculations in both possible space groups for Pb_2_SnO_4_ (Tab. 1 ▸). Our DFT-based calculations reproduce the experimental diffraction data satisfactorily with an overestimation of the unit-cell lengths by <3% due to the well established ‘underbinding’ in DFT-GGA-PBE calculations. The DFT calculations carried out here provide structural and physical parameters in the athermal limit. As has been discussed in the introduction, structurally closely related Pb_2_PbO_4_ undergoes a tetragonal 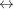 orthorhombic phase transition at 170 K, so it is actually problematic to neglect temperature in DFT studies of this system. The DFT-based calculations gave the same total energy within the numerical accuracy for the orthorhombic and the tetragonal structure.

**Figure 5:**
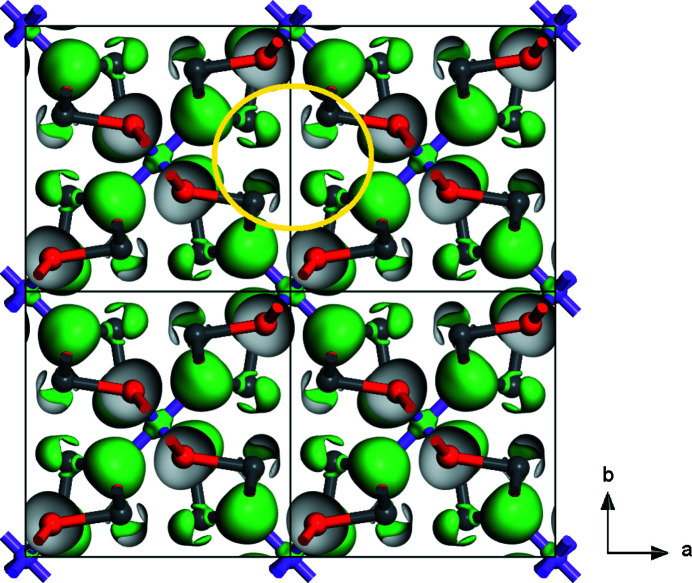
Isosurface of the electron density difference at 0.03 e Å^−1^. Sn atoms, oxygen atoms and lead atoms are represented by violet, red and gray spheres, respectively. The yellow circle highlights a channel running parallel to [001], which hosts the 6*s* ^2^ stereochemically active lone electron pairs of the Pb^2+^ ions, which appear as umbrella shaped surfaces.

Fig. 5 ▸ shows an isosurface of the electron density difference, *i.e.* shows charge accumulation with respect to the electron density obtained by overlapping non-interacting atomic densities.

Clearly discernible are the stereochemically-active 6*s* ^2^ lone electron pairs of the Pb^2+^ ions, which appear in electron density difference isosurfaces as umbrella shaped objects (Friedrich *et al.*, 2010 ▸). These electron pairs are located in the channels of the structure.

### 4.5. High-pressure single-crystal diffraction

We performed high-pressure single crystal X-ray diffraction measurements on Pb_2_SnO_4_ crystals up to 21.0 (4) GPa and solved and refined the crystal structure at numerous pressures (Table 3 ▸). From the single crystal data we found that on pressure increase the unit cell of Pb_2_SnO_4_ is immediately strained and Δ*a* *b* increases from 0.0025 (4) Å at ambient pressure to its maximum of 2.799 (1) Å at 12.4 (3) GPa. After a pressure increase above ∼0.5 GPa the structure of Pb_2_SnO_4_ cannot be described in space group *P*4_2_/*mbc* anymore and only a refinement in space group *Pbam* is satisfactory. At 7.8 (2) GPa (*wR* = 14.0%) and 10.0 (2) GPa (*wR* = 18.5%) the refinements of the structure in the space group *Pbam* yield increasingly worse reliability factors, but attempts to improve the description of the data by changing the structural model to another space group were unsuccessful.

On further pressure increase we observed that Pb_2_SnO_4_ undergoes a phase transition from the orthorhombic space group *Pbam* (No. 55) to *Pnam* (No. 62) between 10 GPa and 12 GPa. At 12.4 (3) GPa (*wR* = 2.3%), the refinement in the high-pressure space group *Pnam* is convincing. We chose the unconventional *Pnam* setting of space group No. 62 in order to facilitate a comparison to the low-pressure structure. A symmetry check with the *PLATON* package (Spek, 2003 ▸) was carried out to confirm the space group symmetry.

After pressure release we measured the same crystal in an opened diamond anvil cell at ambient conditions. The structure refinement shows that the pressure-induced straining of the unit cell and the pressure-induced phase transition is fully reversible on pressure release (Table 3 ▸).

### 4.6. High-pressure powder diffraction

**Figure 6:**
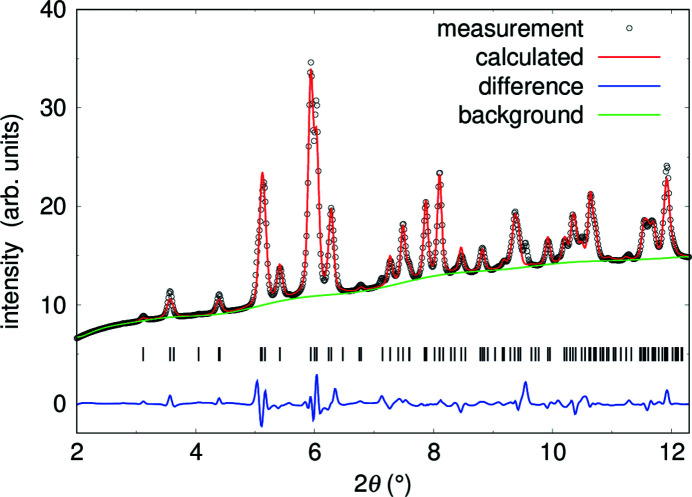
Rietveld refinement of Pb_2_SnO_4_ at 12.0 (2) GPa in space group *Pnam* (*wR* = 2.7%) using λ = 0.2906 Å. Reflection positions are indicated by tickmarks and the residuals between measurement and refinement are shown by the blue line. Diamond and neon reflections were masked before data integration.

The high-pressure X-ray powder data complement the single-crystal data, as they have been measured for pressures up to 50 (1) GPa. We used the results from the single crystal refinement on Pb_2_SnO_4_ as starting model for the refinements of the powder diffraction data. Fig. 6 ▸ shows a Rietveld refinement of data collected at 12.0 (2) GPa, close to the pressure-induced structural phase transition. The refinement was carried out in space group *Pnam*. The anisotropic displacement parameters of the oxygen atoms were constrained to be identical and we applied restraints to ensure that the Sn—O bond distances are ∼2 Å. The high background is caused by diamonds and the pressure transmitting medium. The agreement between the experimental data and the structural model is convincing. The results obtained from the refinements of the powder data are in good agreement with the single crystal data collected up to 21 GPa.

### 4.7. Deformation of the Pb_2_SnO_4_ unit cell

**Figure 7:**
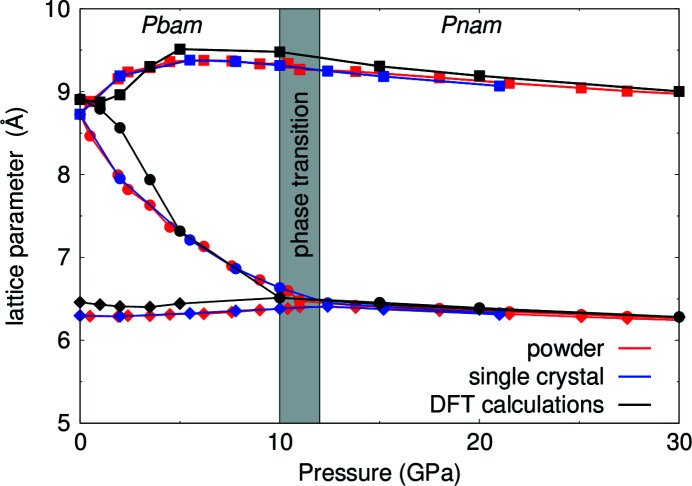
Pressure-dependent behavior of the Pb_2_SnO_4_ lattice parameters, from single-crystal diffraction (blue), powder diffraction (red) and DFT calculations (black). The lattice parameter *a* is shown with black squares, *b* with filled circles and *c* with red diamonds.

Fig. 7 ▸ shows the behavior of the Pb_2_SnO_4_ lattice parameters between ambient conditions and 30 GPa from single crystal and powder diffraction data in comparison to DFT-based calculations. Based on the single crystal and powder diffraction data we observed that the pressure dependence of the unit-cell parameters are very different up to pressures of ∼12 GPa. In this pressure regime, the *a* axis expands on pressure increase, the *b* axis shrinks, and the *c* axis remains essentially unchanged. This observation is supported by the DFT-based calculations.

### 4.8. Description of the high-pressure crystal structure

**Figure 8:**
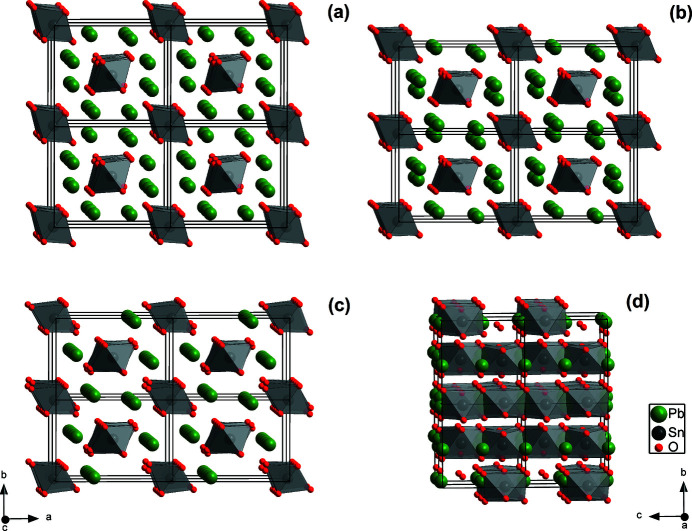
Pb_2_SnO_4_ crystal structure viewed along the *c* axis at 2.0 (1) GPa (*a*), 7.8 (2) GPa (*b*) and 12.4 (3) GPa (*c*). Structure viewed along the *a* axis at 12.4 (3) GPa (*d*). 2 × 2 × 2 supercells are shown and the SnO_6_ octahedra are illustrated in gray.

Fig. 8 ▸ shows the evolution of the crystal structure of Pb_2_SnO_4_ with increasing pressure. The pressure-induced elongation of the *a* axis and the compression in the *b* direction and the concomitant rearrangement of the Pb ions before the phase transition can clearly be observed.

**Figure 9:**
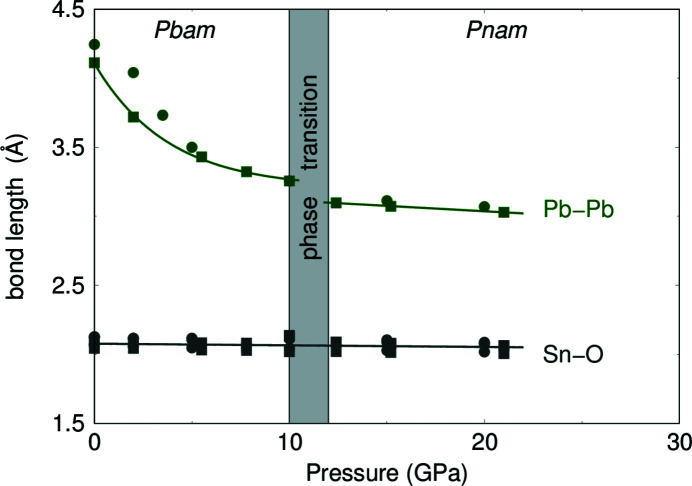
Pb—Pb and Sn—O distances with increasing pressure from single-crystal diffraction (filled square) and DFT-based calculations (filled circle). Lines represent fits to the experimental data points.

The pressure-dependence of the Pb–Pb and Sn—O distances in Pb_2_SnO_4_ are shown in Fig. 9 ▸. We observed that the SnO_6_ octahedra behave as quasi-rigid units in the crystal structure. The Sn—O bond lengths in the SnO_6_ octahedra remains approximately constant (∼2 Å) and are only slightly decreasing with increasing pressure. At ambient conditions the SnO_6_ octahedra have a volume of *V*_SnO_6__ = 11.9 Å^3^ which is decreasing to *V*_SnO_6__ = 11.1 Å^3^ at 21.0 (4) GPa. The distance between the opposite Pb^2+^ ions, forming the channels at ambient conditions (Fig. 1 ▸), is decreasing by 1 Å with increasing pressure from ∼4.1 Å at ambient conditions to ∼3.1 Å at 12.4 (3) GPa. After the phase transition the Pb–Pb distance is almost independent of pressure.

The experimental finding of a phase transition was also supported by DFT-based calculations. The calculations imply, based on the enthalpy difference 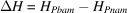 , that the phase transition from space group *Pbam* to *Pnam* occurs just below 10 GPa. The experimentally determined transition pressure and the results from the DFT-based calculations are therefore in good agreement.

**Figure 10:**
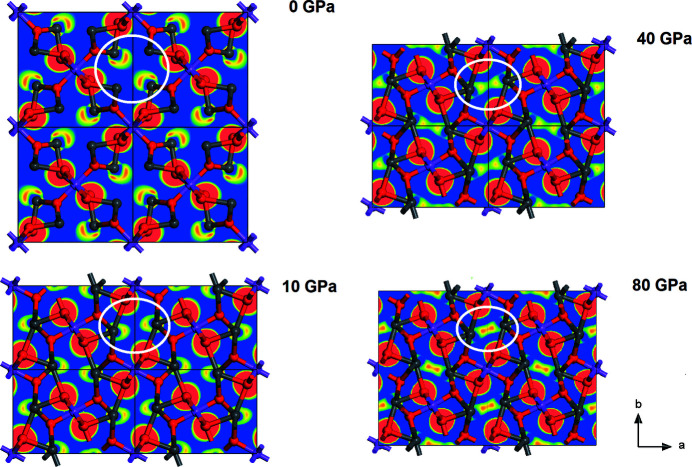
Electron density difference maps obtained by DFT calculations of the pressure induced evolution of the stereochemically active lone electron pairs (highlighted by white circles) show that at high pressures there is a charge accumulation along the Pb—Pb contacts, *i.e.* Pb—Pb bonds have been formed by transferring 6*s* electrons of a Pb atom into empty 6*p* orbitals of a Pb atom <3 Å away. 2 × 2× 2 supercells are shown along the *c* axis.

The DFT calculations show a rather peculiar behavior of the stereochemically active lone electron pairs. While it is well established that such lone electron pairs may persist at high pressures [*e.g.* Friedrich *et al.* (2010 ▸)] Fig. 10 ▸ shows that in Pb_2_SnO_4_ the lone electron pairs overlap on increasing pressure, *i.e.* there is bond formation along the Pb—Pb contacts both within the (001) planes and along the **c** direction.

The formation of Pb—Pb bonds has been discussed earlier [see *e.g.* reviews by Fischer & Power (2010 ▸) and Nagase (2013 ▸)] in diplumbenes, which have Pb—Pb bonds with bond distances of 2.9–4.1 Å. In the present case, the change in the electron density suggests the formation of dative bonds between the Pb^2+^ ions, *i.e.* bonds due to the interaction of the stereochemically active lone electron pairs of the donor atom with unoccupied orbitals of the acceptor atom. A Mulliken population analysis shows that the Pb 6*p* orbitals are filled slightly more on bond formation and the bond population between neighboring Pb^2+^ ions rises up to 0.28 e Å^−3^ at 80 GPa.

### 4.9. Bulk modulus of Pb_2_SnO_4_

**Figure 11:**
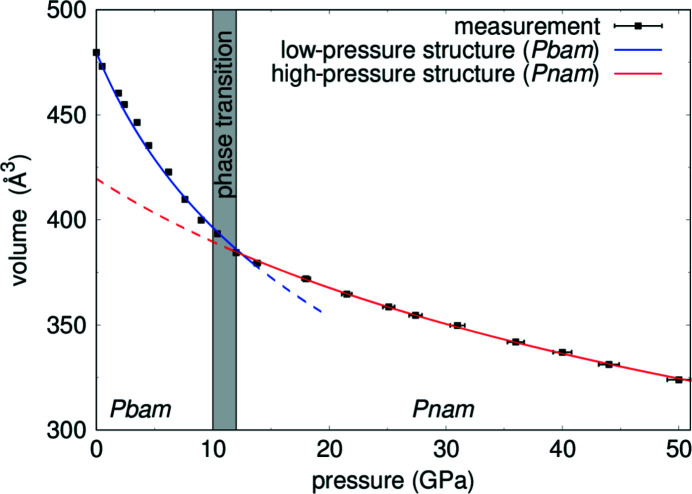
Pressure dependence of the unit-cell volume of Pb_2_SnO_4_ from X-ray powder diffraction data. The second-order Birch–Murnaghan equation of state fitted to the experimental data for the low-pressure phase is shown in blue and for the high-pressure phase in red.

We used the X-ray powder diffraction data to obtain the unit cell volume of Pb_2_SnO_4_ from ambient conditions up to 50 (1) GPa (Fig. 11 ▸). These data sets were used to compute the values of the bulk modulus *K* for the low-pressure phase (*Pbam*) and the high-pressure phase (*Pnam*). We fitted a second-order Birch–Murnaghan equation of state to unit cell volume data up 10.4 (2) GPa for the low-pressure structure. The ambient-pressure volume *V*_0_ was not refined due to the limited data range for this structure and fixed to the volume obtained from ambient-pressure X-ray diffraction. For the high-pressure phase we fitted a second-order Birch–Murnaghan equation of state to the experimental data between 12.0 (2) GPa and 50 (1) GPa, refining the ambient-pressure volume *V*_0_ also. Table 4 ▸ lists the experimental values of *K* for both phases. The bulk moduli of the ambient-pressure phase [*K*_Pbam_ = 36 (2) GPa] and the high-pressure phase (*K*_Pnam_ = 117 (6) GPa) differ significantly.

### 4.10. Resistance measurements

#### 4.10.1. Calibrating the experimental set-up

We performed electrical resistance measurements as a function of pressure in diamond anvil cells. These measurements were calibrated by measuring pure silicon (99.999% purity, Alfa Aesar). At ambient pressure silicon crystallizes in space group 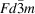 (No. 227) (Cohen & Chelikowsky, 1989 ▸). Between 8 GPa and 12.5 GPa silicon undergoes a phase transition into the β-Sn structure with space group *I*4_1_ (No. 141) (Garg *et al.*, 2004 ▸; Olijnyk *et al.*, 1984 ▸; Weinstein & Piermarini, 1975 ▸; Hu & Spain, 1986 ▸; Gupta & Ruoff, 1980 ▸; Hu *et al.*, 1986 ▸; Yin & Cohen, 1982 ▸; McMahan & Moriarty, 1983 ▸; Chang & Cohen, 1985 ▸; Mizushima *et al.*, 1994 ▸), accompanied by a decrease of the electrical resistance by ∼10^7^ (Garg *et al.*, 2004 ▸; Minomura & Drickamer, 1962 ▸).

**Figure 12:**
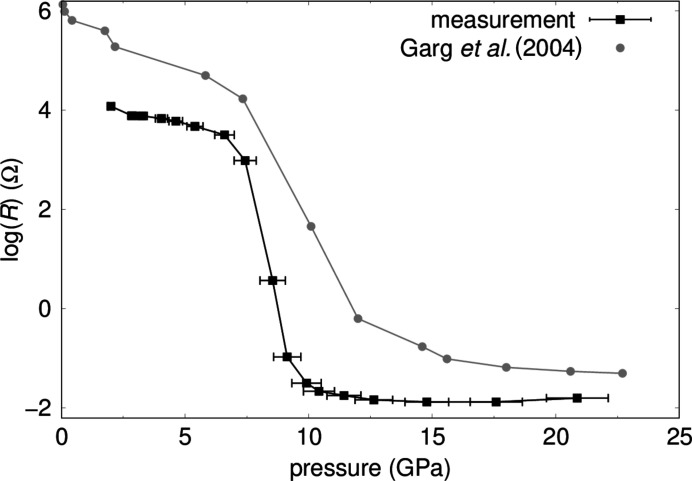
Electrical resistance measurements on Si for calibrating and testing the experimental set-up as function of pressure using the four-point probe method up to 21 (1) GPa in comparison to data of Garg *et al.* (2004 ▸).

Our measurements from ambient conditions to 21 (1) GPa show a change of the resistance of > 10^6^ (Fig. 12 ▸). The resistance decreases by 10^4.5^ within ∼2.5 GPa in the region of the phase transition from Si-I to Si-II and we determined a transition pressure of 8.4 (5) GPa. Garg *et al.* (2004 ▸) measured a decrease of the resistance by 10^4.5^ within ∼5 GPa and a transition pressure of 10.2 GPa using mylar embedded Al_2_O_3_ as pressure transmitting medium. In comparison to Garg *et al.* (2004 ▸) the phase transition occurs in a much narrower pressure-range in our experiments, but at slightly lower pressures. The transition pressure obtained here is in good agreement with the data from Hu *et al.* (1986 ▸) who found that the phase transition occurs at lower pressures of ∼8.5 GPa in a non-hydrostatic environment in comparison to a transition pressure of 11.3 (2)–12.5 (2) GPa for quasi-hydrostatic conditions. Gupta & Ruoff (1980 ▸) found a sensitivity of the Si-I to Si-II phase transition to uniaxial stress and observed a change in the pressure-dependent resistance at 8 GPa by applying uniaxial stress along [111]. In summary, these calibration measurements show that our set-up allows us to accurately measure pressure-induced changes in the resistance but the sample environment is not hydrostatic.

#### 4.10.2. Pressure-dependent resistance of Pb_2_SnO_4_

**Figure 13:**
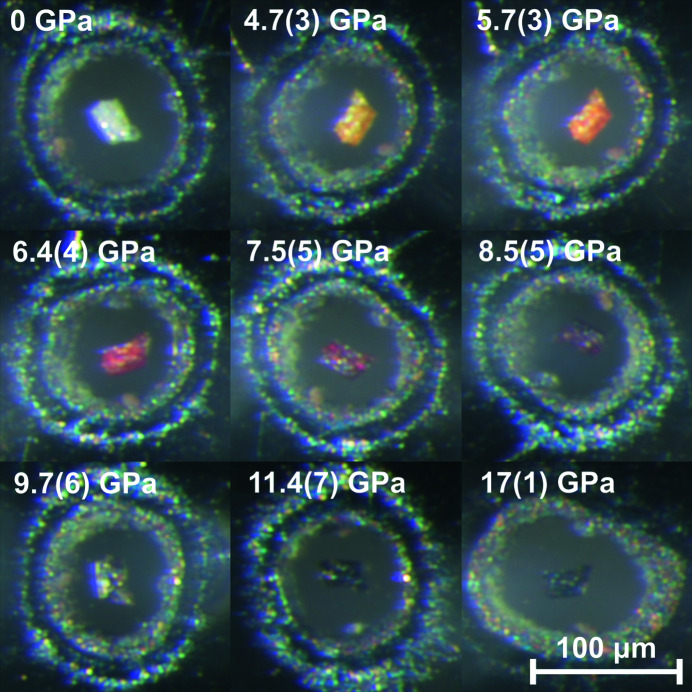
Pressure-dependent color change of Pb_2_SnO_4_ between 0 GPa and 17 (1) GPa in a diamond anvil cell.

Pressure increase leads to a significant color change of Pb_2_SnO_4_ powder and single crystals. At ambient pressure, the crystals are colorless and the powder is lightly yellow. With increasing pressure the light yellow powder at ambient pressure and the single crystals became yellow (∼3 GPa), red (∼6 GPa) and brown (∼8 GPa). On further pressure increase the sample becomes opaque (Fig. 13 ▸). All pressure-induced color changes are fully reversible on pressure release without hysteresis.

The change in color is caused by a change in the absorption of visible light by the sample, indicative of a decrease in the band gap. Electrical resistance measurements were carried out between ambient pressure and 48 (3) GPa using two-point and four-point measurements (Fig. 2 ▸). Below 14.2 (9) GPa the electrical resistance was above the detection limit of our experimental set-up (10 × 10^9^ Ω).

**Figure 14:**
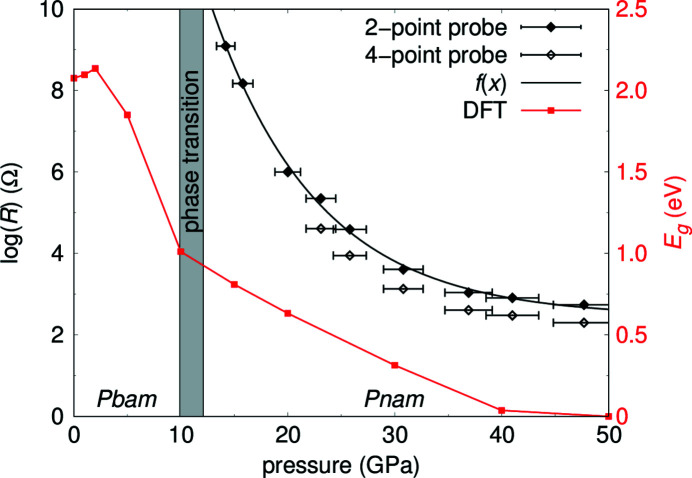
Electrical resistance measurements on Pb_2_SnO_4_ as function of pressure using the two-point probes and the four-point probes method (black). *f*(*x*) shows an exponential fit to the two-point probes measurement. Due to the limitation of the Keithley instrument employed, high resistances could not be observed. The DFT-calculated band gap is shown in red.

The electrical resistance of Pb_2_SnO_4_ decreases by at least six orders of magnitude when the pressure is increased from ambient to ∼40 GPa (Fig. 14 ▸). Due to the limitations of our experimental setup we were not able to determine the resistance of the sample across the structural phase transition. An extrapolation of the resistance to ambient pressure [using *f*(*x*) = *A*_1_·exp(−*x*/*f*_1_) + *y*_0_] suggests a resistivity > 10^14^ Ωm for Pb_2_SnO_4_, similar to insulators such as quartz or corundum. The results from the two-point probes and the four-point probes method are mutually consistent, as it is expected that two-point measurements will yield systematically higher values due to the additional contact resistance of the junction between the sample and the wires.

We calculated the band gap energy *E*_g_ between 0 and 50 GPa by DFT-based calculations and present it together with the electrical resistance measurement (Fig. 14 ▸). The results of these calculations indicate that the band gap is closing between 40 GPa and 50 GPa. However, it is well established that DFT-GGA-PBE calculations underestimate the band gap energy by up to 50% and while the pressure-dependence of the experimentally determined electrical resistance and predicted band gap energy is similar, a quantitative evaluation would require more advanced model calculations. The closing of the band gap is also consistent with the observed pressure-induced change in color.

### 4.11. Raman spectroscopy

**Figure 15:**
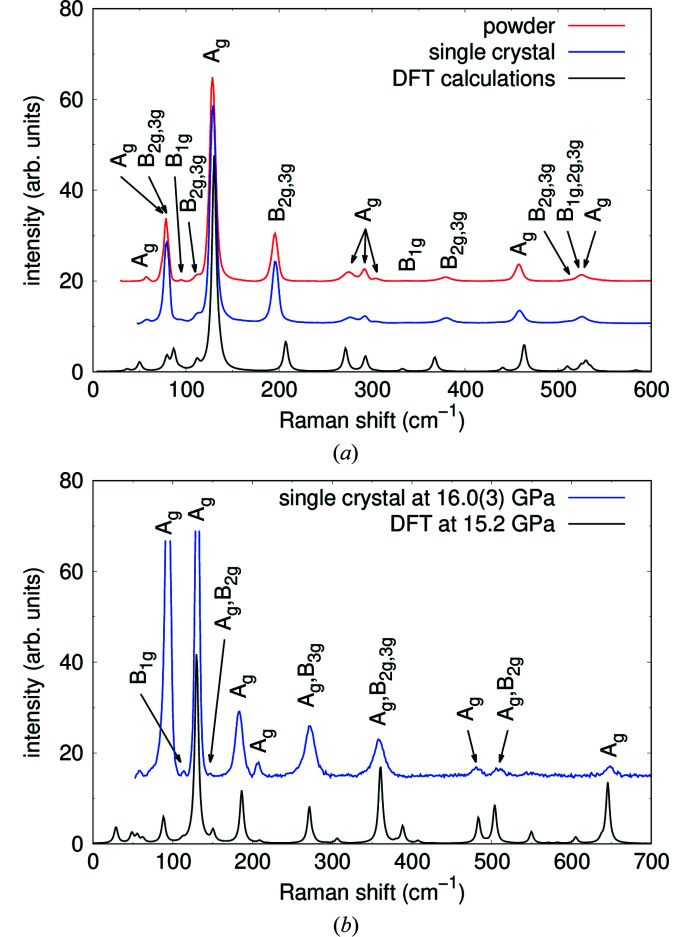
Ambient-pressure Raman spectra of Pb_2_SnO_4_ powder and a single crystal in comparison to results from DFT-based calculations (*a*) and Experimental Raman spectra of a Pb_2_SnO_4_ single crystal at 16.0 (3) GPa in comparison to results from DFT-based calculations at 15.2 GPa carried out in space group *Pnam* (*b*). The frequencies of the calculated Raman spectrum were rescaled by 8%.

Raman spectroscopy was performed on powder samples and on a single crystal. Experimentally determined ambient-pressure Raman spectra of Pb_2_SnO_4_ were satisfactorily reproduced by a theoretical spectrum from DFT-based calculations independent of their synthesis route [Fig. 15 ▸(*a*)]. The theoretical Raman spectra for structures with space group symmetry of *Pbam* or *P*4_2_/*mbc* are almost identical, therefore both reproduced the experimental data. The experimentally obtained Raman spectra are also in good agreement with the measurements from *e.g.* Clark *et al.* (1995 ▸) or Pelosi *et al.* (2010 ▸).

According to a factor group analysis (DeAngelis *et al.*, 1972 ▸) for space group *Pbam* 42 modes (Γ_Raman_ = 12 A_g_ + 12 B_1g_ + 9 B_2g_ + 9 B_3g_) and for space group *P*4_2_/*mbc* 26 modes are Raman active (Γ_Raman_ = 5 A_1g_ + 7 B_1g_ + 5 B_2g_ + 9 E_g_). We assigned irreducible representations to the observed peaks based on the DFT-based calculations in space group *Pbam* [Fig. 15 ▸(*a*)]. Table 5 ▸ shows the Raman shift of the experimental and calculated Raman modes in Pb_2_SnO_4_ together with the corresponding assignment to the irreducible representation from DFT-based calculations for *Pbam*.

Fig. 15 ▸(*b*) shows experimental Raman data at 16.0 (3) GPa in comparison to results from DFT-based calculations at 15.2 GPa. The agreement between the peak positions from the experimental data and DFT-based calculations for the high-pressure structure with *Pnam* space group symmetry is convincing and all experimentally observed Raman peaks can be assigned to their irreducible representations (Γ_Raman_ = 12 A_g_ + 9 B_1g_ + 12 B_2g_ + 9 B_3g_). Table 6 ▸ shows the experimental and theoretical Raman data together with the corresponding assignment to the irreducible representation for the high-pressure space group *Pnam*.

## 5. Discussion and conclusion

The ambient pressure X-ray diffraction data of Pb_2_SnO_4_ can be successfully refined in two space groups (*P*4_2_/*mbc* or *Pbam*) with very similar *R* values. Neither Raman spectroscopy nor DFT calculations can be used to unambiguously distinguish between the two space groups. While the structure of Pb_2_SnO_4_ is undoubtedly very nearly tetragonal, both earlier studies (Gavarri *et al.*, 1981 ▸) and the present experiments lead to the conclusion that there is a small deviation from *P*4_2_/*mbc* and that hence the space group *Pbam* is the preferred choice for the structure of Pb_2_SnO_4_ at ambient conditions.

The high-pressure X-ray diffraction data and DFT-based calculations show a significant pressure-induced distortion from the quasi-tetragonal metric present at ambient conditions with increasing pressure. Pb_2_SnO_4_ with *Pbam* space group symmetry is stable up to 8–10 GPa, when a structural phase transition to a high-pressure structure with space group symmetry *Pnam* occurs. The experimentally observed structural phase transition is consistent with the results from the DFT-based calculations and Raman spectroscopic data. We observed no further phase transition of Pb_2_SnO_4_ up to 50 GPa.

The pressure-induced structural changes lead to a rearrangement of the Pb ions, while the chains formed by the edge-shared SnO_6_ octahedra remain essentially unchanged. The high-pressure phase is stabilized by the formation of Pb—Pb bonds. The presence of the Pb—Pb bonds at high pressures has been inferred from a Mulliken analysis of the electron density obtained from DFT calculations and from electron density difference maps. Our findings are consistent with earlier results on Pb—Pb bonding based on NMR measurements (Gabuda *et al.*, 1999 ▸; Dybowski *et al.*, 2001 ▸), in which it was concluded that the Pb^2+^ 6*p* electron is involved.

The pressure-induced structural changes are accompanied by changes in the physical properties, such as a dramatic change in color and a large change in the resistivity. The experimentally obtained bulk moduli for the low- and high-pressure phase of Pb_2_SnO_4_ differ significantly [*K*_Pbam_ = 36 (2) GPa and *K*_Pnam_ = 117 (6) GPa]. A similar drastic change in the bulk moduli between the low and high-pressure phase has also been observed for the phase transition from phase II [*K*_phase II_ = 20.8 (4) GPa] to phase III [*K*_phase III_ = 98 (3)  GPa] in structurally closely related Pb_2_PbO_4_ (Dinnebier *et al.*, 2003 ▸). The pressure-induced changes in the structural and physical properties are fully reversible on pressure release.

In summary, Pb_2_SnO_4_ was shown to display an interesting high pressure behavior which is associated with a change of the properties of the stereochemically active lone electron pairs present at ambient conditions and the formation of Pb—Pb bonds. Studies to further characterize these bonds are currently underway.

## Supplementary Material

Crystal structure: contains datablock(s) global, Pb2SnO4-P42ombc-P0, Pb2SnO4-Pbam-P0, Pb2SnO4-Pbam-P1, Pb2SnO4-Pbam-P2, Pb2SnO4-Pbam-P3, Pb2SnO4-Pbam-P4, Pb2SnO4-Pnam-P5, Pb2SnO4-Pnam-P6, Pb2SnO4-Pnam-P7. DOI: 10.1107/S205252062001238X/xk5074sup1.cif

Structure factors: contains datablock(s) Pb2SnO4-P42ombc-P0. DOI: 10.1107/S205252062001238X/xk5074Pb2SnO4-P42ombc-P0sup2.hkl

Structure factors: contains datablock(s) Pb2SnO4-Pbam-P1. DOI: 10.1107/S205252062001238X/xk5074Pb2SnO4-Pbam-P1sup3.hkl

Structure factors: contains datablock(s) Pb2SnO4-Pbam. DOI: 10.1107/S205252062001238X/xk5074Pb2SnO4-Pbam-P0sup4.hkl

Structure factors: contains datablock(s) Pb2SnO4-Pbam-P2. DOI: 10.1107/S205252062001238X/xk5074Pb2SnO4-Pbam-P2sup5.hkl

Structure factors: contains datablock(s) Pb2SnO4-Pbam-P3. DOI: 10.1107/S205252062001238X/xk5074Pb2SnO4-Pbam-P3sup6.hkl

Structure factors: contains datablock(s) Pb2SnO4-Pbam-P4. DOI: 10.1107/S205252062001238X/xk5074Pb2SnO4-Pbam-P4sup7.hkl

Structure factors: contains datablock(s) Pb2SnO4-Pnam-P5. DOI: 10.1107/S205252062001238X/xk5074Pb2SnO4-Pnam-P5sup8.hkl

Structure factors: contains datablock(s) Pb2SnO4-Pnam-P6. DOI: 10.1107/S205252062001238X/xk5074Pb2SnO4-Pnam-P6sup9.hkl

Structure factors: contains datablock(s) Pb2SnO4-Pnam-P7. DOI: 10.1107/S205252062001238X/xk5074Pb2SnO4-Pnam-P7sup10.hkl

CCDC references: 2030954, 2030955, 2030956, 2030957, 2030958, 2030959, 2030960, 2030961, 2030962

## Figures and Tables

**Table 1:** Crystallographic data of Pb_2_SnO_4_ at ambient conditions obtained by single-crystal structure solution, Rietveld refinement on powder data and DFT-based calculations in comparison to data from Swanson *et al.* (1972 ▸) and Gavarri *et al.* (1981 ▸)

	This study (tetragonal)			This study (orthorhombic)			Swanson *et al.* (1972 ▸)†	Gavarri *et al.* (1981 ▸)‡ §
	Single crystal	Powder	DFT	Single crystal	Powder	DFT	Powder	Powder
Crystal system	Tetragonal			Orthorhombic			Tetragonal	Orthorhombic
Space group	*P*4_2_/*mbc* (No. 135)			*Pbam* (No. 55)			*P*4_2_/*mbc* (No. 135)	*Pbam* (No. 55)
*a* (Å)	8.7276 (2)	8.7387 (1)	8.9184	8.7288 (3)	8.7425 (2)	8.9298	8.7371 (4)	8.7215 (3)
*b* (Å)	–	–	–	8.7263 (3)	8.7330 (2)	8.9103	–	8.7090 (3)
*c* (Å)	6.2970 (2)	6.3075 (1)	6.4713	6.2969 (2)	6.3068 (2)	6.4715	6.307 (1)	6.2919 (3)
Δ*a* *b* (Å)	0	0	0	0.0025 (4)	0.0095 (3)	0.0195	0	0.0125 (6)
*V* (Å^3^)	479.65 (2)	481.67 (2)	514.71	479.64 (3)	481.51 (4)	514.92	481.46	477.90 (4)
ρ (g cm^−3^)	8.268	8.234	7.705	8.269	8.236	7.702	8.237	8.323
*R*_int_ (%)	4.2	–	–	4.1	–	–	–	–
No. of unique reflections	349	109	–	679	216	–	–	≈ 800
No. of refined parameters	22	24	–	40	33	–	–	47
Refinement								
*R* (%)	2.1	8.8	–	2.5	7.7	–	–	6.9
*wR* (%)	2.3	12.1	–	2.6	10.7	–	–	–
GOF	1.3	2.5	–	1.2	2.2	–	–	–
λ (Å)	0.71073	1.54056	–	0.71073	1.54056	–	1.54056	1.384 (2)

† ICDD No. 00-024-0589.

‡ ICDD No. 01-075-1846.

§ Neutron diffraction.

**Table 2:** Atom positions and anisotropic displacement parameters of Pb_2_SnO_4_ in space groups *P*4_2_/*mbc* and *Pbam* from single-crystal structure refinement Lattice parameters *a*, *b*, *c* in Å and atomic displacement parameters *U*_*ij*_ in Å^2^.

	Atom	Site	*a*	*b*	*c*	*U*_eq_	*U*_11_	*U*_22_	*U*_33_	*U*_12_	*U*_13_	*U*_23_
*P*4_2_/*mbc*	Pb	8*h*	0.16055 (5)	0.14197 (5)	0.5	0.0223 (1)	0.0225 (3)	0.0205 (3)	0.0239 (3)	0.0014 (2)	0	0
	Sn	4*d*	0.5	0	0.75	0.0181 (2)	0.0196 (3)	0.0196 (3)	0.0152 (5)	0.0015 (4)	0	0
	O1	8*h*	0.0973 (8)	0.3750 (8)	1.0	0.020 (2)	0.024 (4)	0.018 (4)	0.019 (4)	−0.003 (3)	0	0
	O2	8*g*	0.3344 (6)	0.1656 (6)	0.75	0.025 (2)	0.027 (3)	0.027 (3)	0.022 (4)	0.003 (3)	0.004 (2)	0.004 (2)
*Pbam*	Pb1	4*g*	0.16058 (5)	0.14191 (5)	0.5	0.0222 (2)	0.0226 (3)	0.0201 (3)	0.0239 (3)	0.0014 (2)	0	0
	Pb2	4*h*	0.35797 (5)	0.33984 (5)	1.0	0.0223 (2)	0.0226 (3)	0.0201 (3)	0.0239 (3)	0.0014 (2)	0	0
	Sn	4*f*	0.5	0	0.7502 (2)	0.0181 (3)	0.0196 (4)	0.0195 (5)	0.0152 (4)	−0.0015 (3)	0	0
	O1	4*h*	0.0979 (8)	0.37653 (8)	1.0	0.021 (3)	0.023 (5)	0.019 (5)	0.021 (5)	−0.002 (4)	0	0
	O2	8*i*	0.3344 (6)	0.16559 (6)	0.752 (1)	0.019 (3)	0.016 (4)	0.023 (5)	0.019 (5)	−0.002 (4)	0	0
	O3	4*g*	0.1264 (6)	0.4381 (6)	0.5	0.025 (2)	0.022 (4)	0.032 (4)	0.022 (4)	−0.003 (3)	−0.003 (3)	0.004 (3)

**Table 3:** Selected crystallographic data of Pb_2_SnO_4_ obtained between ambient conditions and 21 GPa by synchrotron-based single-crystal structure refinements The crystal was colorless at ambient conditions and had approximate dimensions 80 µm × 30 µm × 30 µm.

*p* (GPa)	0.0001†		2.0 (1)	5.5 (1)	7.8 (2)	10.0 (2)	12.4 (3)	15.2 (3)	21.0 (4)
Space group	*P*4_2_/*mbc* (No. 135)	*Pbam* (No. 55)	*Pbam* (No. 55)				*Pnam* (No. 62)		
*a* (Å)	8.7372 (2)	8.7397 (3)	9.1901 (7)	9.3799 (7)	9.3641 (6)	9.3169 (6)	9.2484 (8)	9.1830 (6)	9.0691 (6)
*b* (Å)	–	8.7348 (3)	7.9508 (3)	7.2103 (3)	6.8646 (6)	6.6337 (6)	6.4498 (9)	6.4046 (6)	6.3282 (6)
*c* (Å)	6.3048 (2)	6.3047 (2)	6.2893 (7)	6.3250 (7)	6.3553 (2)	6.3808 (2)	6.4096 (2)	6.3727 (2)	6.3110 (2)
Δ*ab* (Å)	0	0.0049 (4)	1.2393 (8)	2.1696 (8)	2.4995 (9)	2.6832 (9)	2.799 (1)	2.7784 (9)	2.7409 (9)
*V* (Å^3^)	481.30 (2)	481.30 (3)	459.55 (6)	427.77 (6)	408.52 (5)	394.37 (5)	382.33 (6)	374.80 (4)	362.20 (4)
ρ (g cm^−3^)	8.240	8.240	8.630	9.271	9.708	10.056	10.373	10.582	10.950
*R*_int_ (%)	2.0	1.7	1.7	1.5	3.7	7.4	1.4	1.6	1.7
No. of unique reflections	770	1503	1011	898	1085	1004	814	814	813
No. of refined parameters	22	40	40	40	29‡	26‡	38	38	38
*R* (%)	2.0	2.2	2.3	2.1	8.7	13.9	1.9	2.0	2.4
*wR* (%)	2.7	2.9	2.9	2.9	14.0	18.5	2.3	2.7	2.9
GOF	1.8	1.7	1.4	1.6	5.1	6.0	1.2	1.4	1.4

† Opened diamond anvil cell after pressure release.

‡ Anisotropic refinement of atomic displacement parameters was unsuccessful.

**Table 4:** Bulk modulus of the low- and high-pressure structures of Pb_2_SnO_4_ from X-ray powder diffraction

	*K*_exp_(GPa)	*V*_0_ Å^3^)†
*Pbam*	36 (2)	479.64 (3)
*Pnam*	117 (6)	420 (3)

† Constrained to the value obtained from ambient-pressure X-ray diffraction.

**Table 5:** Peak positions (cm^−1^) of selected Raman modes of Pb_2_SnO_4_ from experimental data and DFT-based calculations at ambient conditions together with DFT-based mode assignments to irreducible representation

Experiment	DFT	\Gamma _{{\rm {Raman}}}^{{Pbam}}
56.2	46.0	A_g_
78.3	72.4, 79.7, 79.7	A_g_, B_2g,3g_
94.4	86.6	B_1g_
112.7	102.7, 102.7	B_2g,3g_
128.6	119.5	A_g_
195.2	62.1, 62.9	B_2g,3g_
274.7	249.1	A_g_
292.0	268.6	A_g_
303.9	278.9	A_g_
337.1	305.2	B_1g_
378.8	337.0, 337.0	B_2g,3g_
457.5	425.1	A_g_
508.1	467.8, 467.8	B_2g,3g_
525.3	481.2, 481.2, 486.0, 490.7	B_1g,2g,3g_, A_g_

**Table 6:** Peak positions (cm^−1^) of selected Raman modes of Pb_2_SnO_4_ from experimental [16.0 (3) GPa] data and DFT-based calculations (15.2 GPa) in the high-pressure space group *Pnam* together with DFT-based mode assignments to irreducible representation

Experiment	DFT	\Gamma ^{Pbam}_{\rm Raman}
93.1	81.2	A_g_
113.8	103.1	B_1g_
130.8	119.3	A_g_
146.9	138.1, 138.1	A_g_, B_2g_
183.3	171.1	A_g_
207.1	191.8	A_g_
271.5	248.9, 254.0	A_g_, B_3g_
358.6	329.3, 330.7, 334.0	A_g_, B_2g,3g_
479.7	443.4	A_g_
509.1	462.1, 462.5	A_g_, B_2g_
646.7	592.2	A_g_
